# Characteristics and outcomes of patients hospitalized for infection with influenza, SARS-CoV-2 or respiratory syncytial virus in the season 2022/2023 in a large German primary care centre

**DOI:** 10.1186/s40001-023-01482-z

**Published:** 2023-12-06

**Authors:** C. Quarg, R. A. Jörres, S. Engelhardt, P. Alter, S. Budweiser

**Affiliations:** 1 Department of Internal Medicine III, Division of Pneumology and Respiratory Medicine, RoMed Hospital Rosenheim, Ellmaierstraße 23, 83022 Rosenheim, Germany; 2 Institute and Outpatient Clinic for Occupational, Social and Environmental Medicine, Member of the German Center for Lung Research (DZL), LMU Hospital, Comprehensive Pneumology Center Munich (CPC-M), Ziemssenstraße 1, 80336 Munich, Germany; 3 Department of Medicine, Pulmonary and Critical Care Medicine, Germany, Member of the German Center for Lung Research (DZL), University of Marburg (UMR), Baldingerstraße, 35043 Marburg, Germany; 4 University Hospital Regensburg, Regensburg, Germany

**Keywords:** Hospitalization, PCR test, SARS-CoV-2, RSV, Influenza, Covid-19, Children, Adults, Mortality, ICU admission

## Abstract

**Background:**

In 2022/2023, Influenza A and Respiratory Syncytial Virus (RSV) reappeared in hospitalized patients, which was in parallel to ongoing SARS-CoV-2 infections. The aim of our study was to compare the characteristics and outcomes of these infections during the same time.

**Methods:**

We included patients of all ages with a positive polymerase chain reaction (PCR) test for Influenza A/B, RSV, or SARS-CoV-2 virus hospitalized in the neurological, internal or paediatric units of the RoMed Hospital Rosenheim, Germany, between October 1st 2022 and February 28th 2023.

**Results:**

A total of 906 patients were included (45.6% female; median age 68.0 years; 21.9% Influenza A, 48.2% SARS-CoV-2, 28.3% RSV). Influenza B (0.2%) and co-infections (1.5%) played a minor role. In patients aged ≥ 18 years (*n* = 637, 71%), Influenza A, SARS-CoV-2 and RSV groups differed in age (median 72, 79, 76 years, respectively; p < 0.001). Comorbidities, particularly asthma and COPD, were most prevalent for RSV. 103 patients were admitted to the intensive care unit (ICU) (16.3% Influenza A, 15.3% SARS-CoV-2, 19.2% RSV; *p* = 0.649), 56 died (6.8% Influenza A, 9% SARS-CoV-2, 11.1% RSV; *p* = 0.496). RSV showed the highest frequencies of low-flow oxygen supplementation for admission and stay. Differences in the length of stay were minor (median 7 days). Conversely, in patients aged < 18 years (*n* = 261, 28,8%), 19.5%, 17.6% and 60.2% were in the Influenza A, SARS-CoV-2 and RSV groups, respectively; 0.4% showed Influenza B and 2.3% co-infections. 17 patients were admitted to ICU (3.9% Influenza A, 9.6% RSV, 0% SARS-CoV-2); none died. RSV showed the highest frequencies of high- and low-flow oxygen supplementation, SARS-CoV-2 the lowest.

**Conclusion:**

When comparing infections with Influenza, SARS-CoV-2 and RSV in the winter 2022/2023 in hospitalized adult patients, rates of ICU admission and mortality were similar. RSV showed the highest frequencies of obstructive airway diseases, and of oxygen supplementation. The latter was also true in children/adolescents, in whom RSV dominated. Thus, in the situation of declining importance of SARS-CoV-2, RSV showed a disease burden that was relatively higher than that from Influenza and SARS-CoV-2 across ages, and this might be relevant for the seasons coming.

**Supplementary Information:**

The online version contains supplementary material available at 10.1186/s40001-023-01482-z.

## Background

Lower respiratory tract infections are a common cause of hospitalization and significantly contribute to morbidity and mortality particularly in young children and older adults [1, 2]. Among the viral causes, Respiratory Syncytial Virus (RSV) and various strains of Influenza, particularly Influenza A, have been most prominent for a long time [3–5]. Starting in early 2020, SARS-CoV-2 dominated this type of infection, while the role of previously relevant viruses became minor or even disappeared, although this impression might have been favoured by the practice of regular testing for SARS-CoV-2. Those papers that addressed other viruses during this time confirmed the predominant role of SARS-CoV-2 [6, 7]. Consequently, nearly all comparisons of the disease burden between viruses were based on historical data [8–10].

Starting in the summer of 2022, the formerly prevalent viruses reappeared, e.g. in terms of Influenza or in terms of RSV, particularly in children [11–13], and a very recent study used data from the same season 2022/2023 to compared Influenza and SARS-CoV-2 [14]; RSV was not included in this analysis. As shown by many studies, the risk from SARS-CoV-2 declined over time, possibly due to the prevalence of less harmful variants [15–17], improved immunity due to infection or vaccination, and advances in the management of Covid-19 patients. In addition, the population at risk may have changed over time. Thus, in-time comparisons are of great value.

The re-appearance of infections with previously prevalent viruses renders it possible to compare disease burden, characteristics of patients at risk, treatment and outcome under comparable conditions. Hospitalized patients are of interest not only due to their disease severity but also, because availability, validity and comparability of data are probably higher than for non-hospitalized patients.

Based on these considerations, we studied infections with Influenza A/B, RSV and SARS-CoV-2 in recent time (October 2022 to February 2023) in patients of a large primary care hospital located in a region known as former Covid-19 hotspot [18, 19] covering the full range of age from newborns to very old individuals.

## Methods

### Study population

In this retrospective study, the initial population (*n* = 1175) comprised patients of all ages with a positive PCR for Influenza A/B, RSV or SARS-CoV-2, who were hospitalized at the RoMed Hospital Rosenheim, Germany, between October 1st 2022 and February 28th 2023. From these, we only included patients admitted to the internal medicine, neurology and paediatric units and excluded patients of other areas, especially gynaecological and surgical units, based on the consideration that the infection was not the primary cause for hospitalization. Initially questionable PCR tests were clarified by follow-up testing. During the time of the study, testing for SARS-CoV-2 upon admission was still obligatory for hospitalized patients, whereas that for Influenza and RSV by tests combined with SARS-CoV-2 was performed in case of clinical hints that these viruses may be present. In case of readmission with the same viral infection within 4 weeks, only the first admission was considered. The study was approved by the Ethical Committee of the University of Regensburg (#23.3289-104).

### Assessments

The presence of Influenza A/B, RSV or SARS-CoV-2 infection was determined by PCR tests performed upon admission or during hospitalization. The following PCR test kits were used: Cepheid^®^ Xpert^®^ Xpress SARS-CoV-2 and Cepheid^®^ Xpert^®^ Xpress SARS-CoV-2/Flu/RSV (XP3SARS-COV2-10, Cepheid GmbH, Krefeld, Germany), BD SARS-CoV-2/Flu with BD MAX™ System (445011, BD Becton Dickinson GmbH, Sparks, Maryland, USA), Rhonda player Point-of-care analyser for SARS-CoV-2 (SD003-02-020-A01, Spindiag GmbH, Freiburg i. Br., Germany).

The relevant information was extracted from the medical records comprising age, sex, body mass index (BMI), comorbidities, symptoms (cough, dyspnoea, fatigue, fever, diarrhoea, nausea) upon admission, and vital signs upon admission (heart rate, respiratory rate, body temperature, blood pressure, oxygen saturation (SpO_2_)). Furthermore, blood gas parameters (pH, arterial pressures of oxygen (pO_2_) and carbon dioxide (pCO_2_)) were collected, as well as laboratory parameters upon admission (glomerular filtration rate estimated via creatinine (eGFR), leukocyte count, C-reactive protein (CRP), haemoglobin, lactate dehydrogenase (LDH), troponin, N-terminal pro b-type natriuretic peptide (NT-proBNP), D-dimers). The assessment of treatment modalities included invasive mechanical ventilation (MIV), non-invasive ventilation (NIV), and high-flow or low-flow oxygen supplementation.

### Outcomes

As primary outcomes, we defined admission to an intensive care unit (ICU) and in-hospital mortality. Secondary outcomes comprised invasive or non-invasive ventilation (NIV), or high-flow or low-flow oxygen supplementation, and the length of the hospital stay. Low-flow means application via Venturi mask/nasal cannula using flow rates of 2–4 L/min in the great majority of cases, in rare cases up to 15 L/min. High-flow means application via nasal cannula (HFNC) or Venturi mask with flow rates from 30 up to 60 L/min, depending on the patient’s compliance.

### Statistical analysis

Numbers and percentages, or median values and quartiles were computed to describe the data. To compare the types of infection, Chi-square statistics and Fisher’s exact test, or the Kruskal–Wallis test were used, depending on the type and structure of the data. Post hoc comparisons were performed using the Mann–Whitney U-test with Bonferroni correction. Moreover, we used multiple binary logistic regression to examine the relationships between patients’ characteristics or treatments and outcomes. Receiver operator characteristics (ROC) analysis was performed for the primary outcomes to determine cut-off values for continuous variables. As tests for SARS-CoV-2 were obligatory and those for Influenza and RSV performed on demand in case of clinical hints, we performed a sensitivity analysis for the primary outcomes based on an evaluation of all patients’ files. For this purpose, patients were categorized according to the evidence that their infection was the likely cause of their hospital stay, or secondary. The statistical software SPSS (version 26, IBM Corporation, Armonk, NJ, USA) was used for data analysis. The level of statistical significance was assumed at *p* < 0.05.

## Results

### Anthropometric data and distribution of infections

Of 921 cases from the internal medicine, neurology and paediatric units, 906 patients (413 women (45.6%), 493 men (54.4%)) remained eligible for analysis, since they were not admitted a second time with the same virus within 4 weeks. The median age (quartiles) of the study population was 68.0 (0.4; 81.3) years, the BMI 25.7 (22.9; 29.3) kg/m^2^. The distribution of sex and age according to infection groups is given in Table 1. The majority of patients were infected with SARS-CoV-2, while the second most common virus was RSV, followed by Influenza A. Due to the fact, that the group of patients with Influenza B comprised only 2 patients and the groups with combined infections were very small, all subsequent statistical comparisons were limited to the groups of Influenza A, SARS-CoV-2 and RSV.

**Table 1:** Baseline characteristics stratified for the type of infection

Viral infection	n = 906 **	n (< 18 years)*	Sex (m/f)*	Age (years)	BMI (kg/m^2^)
Influenza A	198 (21.9%)	51 (25.8%)	106/92 (53.5%/46.5%)	64 (15; 78)	26.0 (23.2; 29.8)
Influenza B	2 (0.2%)	1 (50%)	1/1 (50%/50%)	22 (n.d.)	n.d
SARS-CoV-2	437 (48.2%)	46 (10.5%)	243/194 (55.6%/44.4%)	77 (61; 84)	25.5 (22.8; 29.1)
RSV	256 (28.3%)	157 (61.3%)	136/120 (53.1%/46.9%)	2 (0.4; 70)	26.0 (22.4; 29.3)
Influenza A + SARS	4 (0.4%)	0 (0%)	3/1 (75%/25%)	83 (75; 86)	25.0 (n.d.)
Influenza A + RSV	6 (0.7%)	3 (50%)	2/4 (33.3%/66.7%)	26 (4; 59)	24.1 (n.d.)
Influenza B + RSV	1 (0.1%)	1 (100%)	1/0 (100%/0%)	n.d	n.d
SARS + RSV	2 (0.2%)	2 (100%)	1/1 (50%/50%)	0.15 (n.d.)	n.d

Numbers (percentages) and median values (quartiles) are given. * Percentages refer to infection group (rows). BMI = body mass index. ** Percentages refer to total group of 906 patients. n.d. = not determined

**Fig. 1:**
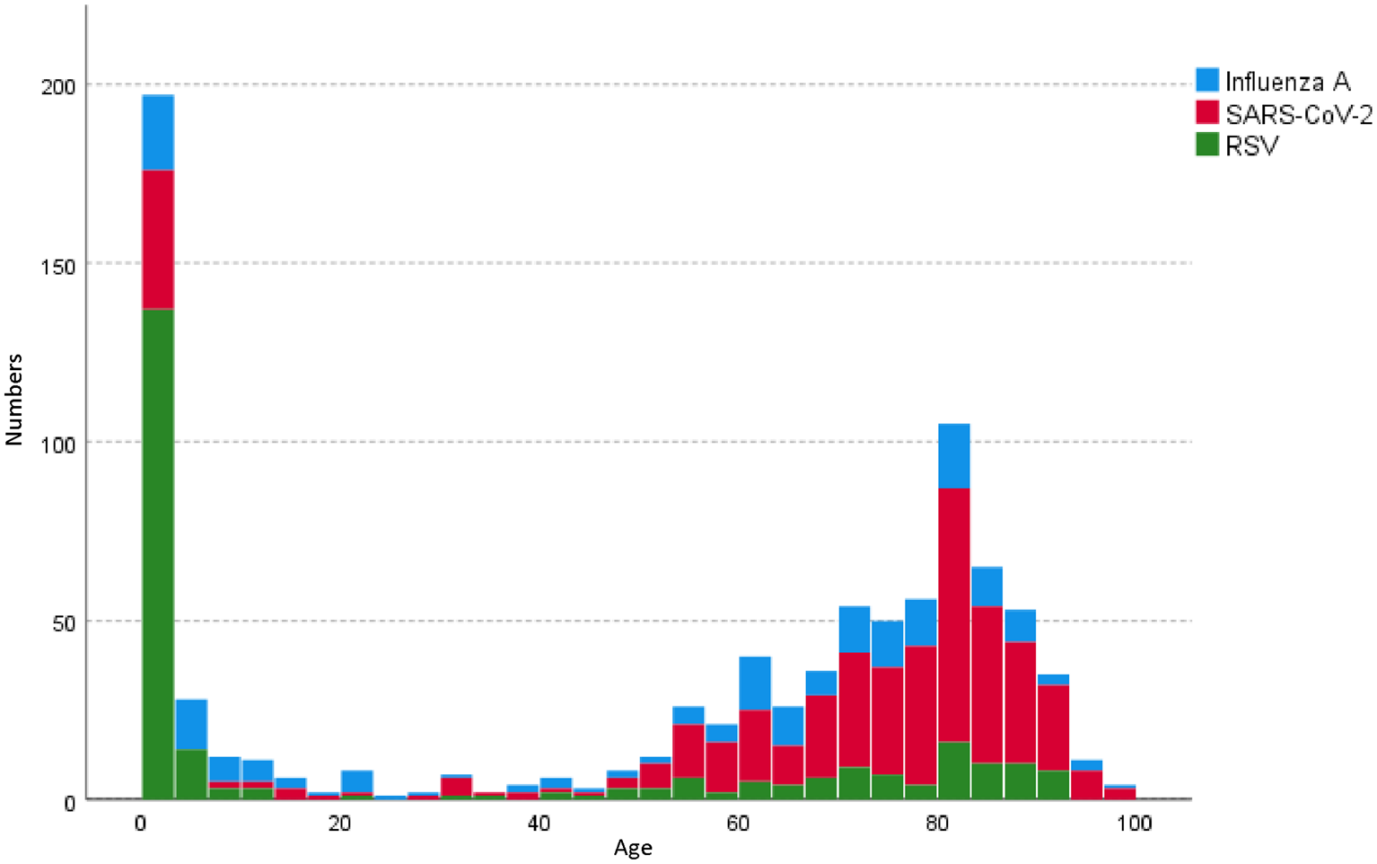
Age distribution in the three major infection groups (see Table 1) that are indicated by different colours. Absolute numbers for each age bin are given

Regarding BMI and sex, there were no significant differences between the three groups. For age, however, all three groups were different from each other (*p* < 0.001 each). Age distribution is illustrated in Fig. 1, showing two distinctive peaks. When restricting the analysis to patients with age ≥ 18 years, age again differed between the three groups (*p* < 0.001), with median values (quartiles) of 72 (62; 80) for Influenza A, 79 (68; 84) for SARS-CoV-2, and 76 (63; 85) years for RSV. In this case, age was only different between Influenza A and SARS-CoV-2 (*p* < 0.001), whereas the RSV group did not significantly differ from the other two groups. In patients of age < 18 years, age also differed between groups (p < 0.001), with median values (quartiles) of 3.8 (1.9; 8.4) for Influenza A, 0.51 (0.16; 1.87) for SARS-CoV-2, and 0.49 (0.17; 1.92) years for RSV. The SARS-CoV-2 and the RSV group were not different from each other, but age of both significantly differed from age of Influenza A patients (*p* < 0.001 each).

### Comorbidities

In patients aged ≥ 18 years, heart failure, rheumatic disease, COPD, and a state of immunosuppression were most often present with RSV. In SARS-CoV-2 patients, asthma and COPD showed the least prevalence, peripheral arterial disease (PAD) the highest. Chronic kidney disease (CKD) was least prevalent in Influenza patients. If expressed as sum of the comorbidities given in Table 2, this sum was significantly greater in RSV patients compared to the other two groups (*p* ≤ 0.003 each).

**Table 2:** Distribution of comorbidities of patients aged ≥ 18 years

Prevalence of comorbidities of patients aged ≥ 18 years				
	Influenza A	SARS-CoV-2	RSV	p value
n	147	391	99	–
Hypertension	81 (55.1%)	254 (65.0%)	64 (64.6%)	0.098
Peripheral arterial disease (PAD)	4 (2.7%)	38 (9.7%)	5 (5.1%)	0.014
Heart failure	25 (17.0%)	84 (21.5%)	34 (34.3%)	0.005
Coronary artery disease (CAD)	42 (28.6%)	99 (25.3)	36 (36.4%)	0.088
Diabetes mellitus type 2	30 (20.4%)	112 (28.6%)	31 (31.3%)	0.096
COPD	30 (20.4%)	43 (11.0%)	30 (30.3%)	< 0.001
Asthma	25 (17.0%)	20 (5.1%)	18 (18.2%)	< 0.001
Other lung disease*	7 (4.8%)	19 (4.9%)	5 (5.1%)	0.995
Chronic kidney disease (CKD)	17 (11.6%)	86 (22.0%)	23 (23.2%)	0.017
Active malignant disease**	12 (8.2%)	56 (14.3%)	11 (11.1%)	0.142
Rheumatic disease	6 (4.1%)	18 (4.6%)	11 (11.1%)	0.028
Depression	9 (6.1%)	31 (7.9%)	10 (10.1%)	0.521
Dementia	18 (12.2%)	49 (12.5%)	11 (11.1%)	0.928
State of immunosuppression	19 (12.9%)	50 (12.8%)	22 (22.2%)	0.049
Number of comorbidities***	2 (1; 3)	2 (1; 4)	3 (2; 4)	< 0.001

Numbers (percentages) are given, for the sum of all comorbidities median values (quartiles). Statistical comparisons were performed with the Chi-square statistics or the Kruskal–Wallis test. COPD = chronic obstructive pulmonary disease. *except malignant diseases of the lung. **within the last 5 years. *** count of all comorbidities listed in the table

To clarify, to which extent the differences in prevalence could be attributed to the differences in age, we performed logistic regression analyses with age and the three types of infections as predictors, and the comorbidities as outcomes. Age was significantly (*p* < 0.05 each) associated with hypertension, PAD, heart failure, coronary artery disease (CAD), diabetes mellitus type 2, asthma, CKD, and dementia but all comorbidities that showed a significant unadjusted difference between infections (Table 2) remained significantly (*p* < 0.05 each) linked to the different infections, suggesting virus-specific, age-independent risk profiles. In patients of age < 18 years, comorbidities were not analysed due to lack of data.

### Primary outcomes

The distribution of ICU treatment and in-hospital mortality for all patients and all infections is shown in Additional file 2: Table S1. Neither the frequency of ICU admission (*p* = 0.974) nor that of in-hospital mortality (*p* = 0.109) differed significantly between the three groups Influenza A, SARS-CoV-2 or RSV. To account for the differences in clinical characteristics, the age groups < 18 years and ≥ 18 years were then analysed separately (Table 3). In adults, the three major infection groups again showed no significant differences regarding ICU admission (*p* = 0.649) or in-hospital mortality (*p* = 0.496). Of the younger patients, all survived, and it appeared that ICU admission was more frequent in the RSV group, but due to low case numbers the Chi-square statistics was of limited value.

**Table 3:** Outcome data of the three major infection groups stratified according to age

Age group	< 18 years			≥ 18 years		
Infection	n	ICU admission	Mortality	n	ICU admission	Mortality
Sample size	254	17 (6.7%)	0 (0%)	637	103 (16.2%)	56 (8.8%)
Influenza A	51	2 (3.9%)	0 (0%)	147	24 (16.3%)	10 (6.8%)
SARS-CoV-2	46	0 (0%)	0 (0%)	391	60 (15.3%)	35 (9.0%)
RSV	157	15 (9.6%)	0 (0%)	99	19 (19.2%)	11 (11.1%)

Numbers (percentages) are given. For the results of statistical comparisons, see text. Mortality refers to in-hospital mortality. ICU = intensive care unit

### Treatment characteristics

Table 4 provides data on the treatment characteristics of patients aged ≥ 18 years. The length of the hospital stay differed between the three groups (*p* = 0.021), with a significant (*p* = 0.033) difference between SARS-CoV-2 and RSV. There were no significant differences regarding the other durations. The frequencies of NIV, low-flow oxygen supply during the hospital stay and oxygen supply upon admission differed significantly between the three groups (*p* < 0.001 each), whereby the highest percentages were observed in the RSV group.

**Table 4:** Treatment characteristics of patients of age ≥ 18 years for the three major infection groups

Treatment characteristics of patients aged ≥ 18 years				
	Influenza A	SARS-CoV-2	RSV	p value
n	147	391	99	–
Length of hospital stay, days	7 (4; 10)	7 (4; 12)	6 (3; 9)	0.021
Intensive care unit				
Frequency	24 (16.3%)	60 (15.3%)	19 (19.2%)	0.649
Length of stay, days	2.7 (1.0; 6.7)	2.8 (0.9; 7.1)	2.1 (1.2; 5.6)	0.981
Mechanical invasive ventilation				
Frequency	8 (5.4%)	17 (4.3%)	3 (3.0%)	0.662
Duration, hours	33.6 (11.5; 138.0)	157.1 (20.8; 298.1)	57.7 (n.d.)	0.439
Non-invasive ventilation				
Frequency	10 (6.8%)	11 (2.8%)	12 (12.1%)	0.001
Duration, hours	7.9 (2.8; 60.5)	13.3 (7.5; 47.2)	28.1 (9.6; 56.5)	0.460
Oxygen supplementation				
High-flow during stay	13 (8.8%)	25 (6.4%)	9 (9.1%)	0.486
Low-flow during stay	99 (67.3%)	227 (58.1%)	82 (82.8%)	< 0.001
Upon admission	46 (31.3%)	102 (26.1%)	46 (46.5%)	< 0.001

Numbers (percentages) are given. Statistical comparisons were performed with the Chi-square statistics and the Kruskal–Wallis test. Durations refer to the subgroups of patients in whom the respective treatment was applied

Table 5 shows analogous data for patients aged < 18 years. The length of the hospital stay differed between the three groups (p < 0.001), with significant differences between RSV and SARS-CoV-2 as well as Influenza A (p ≤ 0.003 each). There were no significant differences regarding the other durations. The frequencies of high-flow and low-flow oxygen supply during the hospital stay also significantly differed between groups (*p* ≤ 0.004 each), whereby the highest percentages occurred in the RSV group.

**Table 5:** Treatment characteristics of patients of age < 18 years for the three major infection groups

Treatment characteristics of patients aged < 18 years				
	Influenza A	SARS-CoV-2	RSV	*p* value
n	51	46	157	–
Length of hospital stay, days	2 (1; 5)	2 (1; 3)	4 (2; 6)	< 0.001
Intensive care unit				
Frequency	2 (3.9%)	0 (0%)	15 (9.6%)	0.05
Length of stay, days	4.9 (n.d.)	n.d	3.7 (2.4; 7.8)	0.881
Mechanical invasive ventilation				
Frequency	0 (0%)	0 (0%)	3 (1.9%)	0.391
Duration, hours	n.d	n.d	108.8 (n.d.)	–
Non-invasive ventilation				
Frequency	0 (0%)	0 (0%)	3 (1.9%)	0.391
Duration, hours	n.d	n.d	66.9 (n.d.)	–
Oxygen supplementation				
High-flow during stay	3 (5.9%)	1 (2.2%)	29 (18.5%)	0.004
Low-flow during stay	15 (29.4%)	0 (0%)	114 (72.6%)	< 0.001
Upon admission	2 (3.9%)	0 (0%)	11 (7.0%)	0.151

Numbers (percentages) and median values and quartiles are given. n.d. = not determined. Statistical comparisons were performed with the Chi-square statistics but partially have to be considered only as hints due to the low case numbers. Durations refer to the subgroups of patients in whom the respective treatment was applied

As the group of very young patients seemed of particular interest, we analysed data of children aged < 3 years separately (Additional file 2: Table S2). The majority had RSV infection; these patients again showed the longest duration of their hospital stay and the highest percentage of oxygen therapy, either low-flow or high-flow.

### Prevalence of symptoms

Symptoms were analysed only for patients aged ≥ 18 years. The prevalence of cough, dyspnoea and fever showed significant differences between the three major types of infection (*p* < 0.001 each), with low values for cough and dyspnoea in SARS-CoV-2, high values for cough and dyspnoea in RSV, and a high value of fever in Influenza A (Additional file 2: Table S3). To account for a possible dependence on age, again logistic regression analyses were performed including age and the type of infection as predictors, and each symptom as outcome. The prevalence of nausea decreased with increasing age, while that of fatigue increased but the unadjusted differences between virus type (Additional file 2: Table S3) remained significant, suggesting virus-specific, age-independent patterns of symptoms.

### Vital parameters, arterial blood gas and laboratory parameters upon admission

For patients ≥ 18 years, vital parameters upon admission are given in Additional file 2: Table S4. All of them, except systolic blood pressure, differed significantly (*p* < 0.05 each) between infection groups. According to post hoc comparisons, SARS-CoV-2 and RSV differed regarding respiratory rate, heart rate, oxygen saturation and diastolic blood pressure (*p* < 0.05 each). For heart rate, temperature and oxygen saturation, there were also significant differences between Influenza A and SARS-CoV-2 but we never observed significant differences between the Influenza A and RSV group. pO_2_ did not significantly differ between groups, but pCO_2_ and pH did. Regarding pH, the SARS-CoV-2 group showed higher values than the other two groups; regarding pCO_2_, values were highest in the RSV group (*p* < 0.05 each). Among laboratory parameters, only eGFR, CRP and D-dimers were significantly different between groups (*p* < 0.05 each) and are shown in the table. Specifically, eGFR and CRP differed between Influenza A and SARS-CoV-2 groups, both with higher values for Influenza A. D-dimers differed between SARS-CoV-2 and RSV, with higher values in SARS-CoV-2.

### Risk factors for ICU admission and in-hospital death

The descriptive results given above showed similarities between the three infections, but also pointed towards differences. As some of the risk factors, such as age or comorbidities, were linked to each other, we performed multiple logistic regression analyses with the aim to identify the statistically independent predictors of ICU admission or in-hospital death. The initial choice of variables was guided by their potential relevance for the outcomes; in the final set we eliminated all predictors with *p*-values of 0.10 or higher. However, the three infection categories (Influenza A, SARS-CoV-2, RSV) were always kept as predictors irrespective of statistical significance, with Influenza A as reference, in order to compare their impact with that of other predictors. The approach followed by us is outlined in the Supplement in detail Additional file 1: Figure S1.

**Fig. 2:**
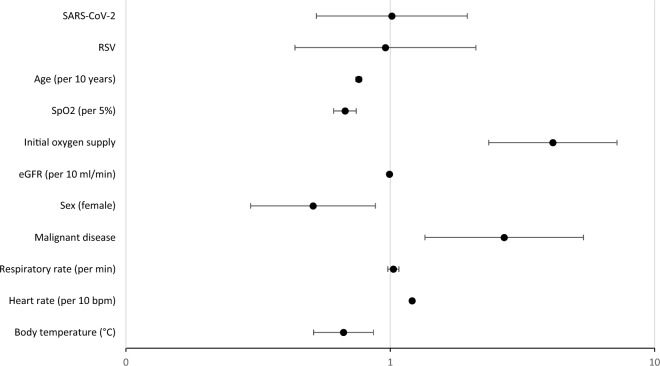
Odds ratios and 95% confidence intervals for the predictors of ICU admission—Influenza A served as reference

Regarding ICU admission, the results and odds ratios of associations are illustrated in Fig. 2, indicating that oxygen supply upon admission was the most consistent, strongest (*p* < 0.001) predictor of subsequent ICU admission for all three viruses, with an odds ratio (95% CI) of 3.88 (2.35; 6.42). Age, sex, active malignant diseases, heart rate, body temperature and oxygen saturation upon admission were additional predictors, while the type of infection was not significantly associated with ICU admission, in line with the unadjusted comparisons.

**Fig. 3:**
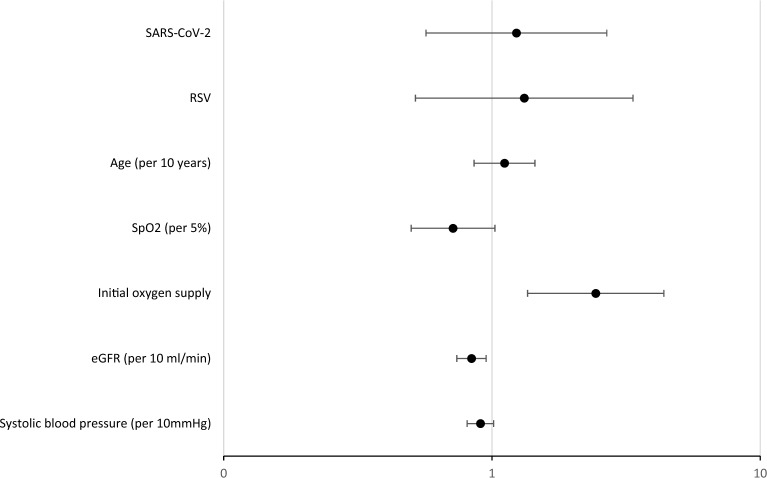
Odds ratios and 95% confidence intervals for the predictors of in-hospital mortality—Influenza A served as reference

Regarding in-hospital mortality, odds ratios are shown in Fig. 3. eGFR and initial oxygen supply were robust and significant predictors (p < 0.05 each), whereas initial oxygen saturation and systolic blood pressure showed only a tendency. The type of infection was not significant, in accordance with the unadjusted comparisons.

### Sensitivity analysis

For this purpose, we identified patients, in whom the infection was considered to have been the likely cause of their hospital stay. In patients < 18 years of age, only 24 of 254 were excluded as having incidental positive tests for any of the three viruses. The numbers of ICU admission were unchanged. Regarding in-hospital mortality, there were also no changes as this was already zero.

In patients of age ≥ 18 years, 202 of 637 were excluded (Additional file 2: Table S5). More SARS-CoV-2 patients were excluded compared to the other two viruses. The relative frequencies of ICU admission did not change significantly by exclusion, neither for the total group, nor for the three infection groups separately. In contrast, overall in-hospital mortality became lower (5.0% versus 10.6%, *p* = 0.023), but again without significant difference within the three infection groups, probably due to the low number of deceased patients.

## Discussion

In this study, the clinical characteristics and outcomes of patients hospitalized with Influenza A, SARS-CoV-2 or RSV between October 1st 2022 and February 28th 2023 were analysed to assess differences and similarities. Its strength is the simultaneous collection of most recent data. This was enabled by the fact that the high numbers of the major infections allowed a direct comparison, whereas nearly all previous studies relied upon data from different seasons. In children and adolescents, the comparatively high disease burden from RSV that is well known from the past was essentially confirmed. In adults, the three infections had a similar impact on the relative risk for ICU admission and in-hospital mortality; although differences were not statistically significant, values were highest for RSV. In terms of clinical and treatment characteristics, respiratory symptoms, a history of obstructive airway disease, non-invasive ventilation and low-flow oxygen supply were most frequent for RSV. Taken together, our findings indicate that in the season 2022/2023 all three infections played an important role in hospitalized adult patients. In particular, they underline that RSV is worth of further attention not only in children.

In the beginning of 2020, SARS-CoV-2 became the dominant respiratory viral infection worldwide with regard to hospitalization rate and mortality [20]. During this time, the incidence of other respiratory viruses, particularly RSV and Influenza, appeared to decrease drastically [6, 7]. Since then, however, the impact of SARS-CoV-2 has declined, as reflected in a reduction of mortality [14], which was already visible when comparing the first and second wave of Covid-19 in 2020/2021 [18]. In parallel, respiratory viruses including Influenza and RSV reappeared, particularly RSV in children [11–13]. We confirmed this in both young and adult patients for the season 2022/2023. The large number of hospitalized patients enabled a comparison in the same population at the same time. The season 2022/2023 also provided the most recent information on Influenza, SARS-CoV-2 and RSV, which might be relevant for predicting future developments.

Previous comparisons between SARS-CoV-2, Influenza and RSV based on data from different times indicated both similarities and differences. A recent study from Germany [21], that examined outcomes and patients’ characteristics in the three types of infection between 2017 and 2020, found a higher risk of ICU admission and hospital death for RSV compared to Influenza A, whereas the risk for SARS-CoV-2 was even higher. In addition, the RSV patients were more likely to have COPD or CKD. Our findings from 2022/2023, i.e. a much later time, confirmed the high risk from RSV particularly in COPD patients, but did not find an elevated risk from SARS-CoV-2.

Another study from Switzerland compared the outcomes of patients infected with Influenza from 2018 to 2022 and with SARS-CoV-2 in 2022 [22]. There was no difference in the risk of ICU admission, but mortality was higher for SARS-CoV-2 than for Influenza (7% vs. 4.4%). Similar observations regarding SARS-CoV-2 versus Influenza were made in a recent study on the outcomes of the last season from October 2022 to January 2023 [14]. Again, SARS-CoV-2 was associated with higher mortality risk than Influenza, but the results also showed that the difference had decreased compared to 2020 (SARS-CoV-2: 6% versus 17–20% in 2020, Influenza: 3.7% versus 3.8% in 2020).

These data again underline that the mortality of Covid-19 patients decreased over time. Our data agree with this regarding ICU admission and length of ICU stay in adults. They also showed a difference between the two infections regarding mortality, irrespective of the fact, whether all patients were included or only those, in whom the infection was considered to be the likely cause of their hospital stay. The differences in mortality between the total group and the subgroup were, however, considerable (see Additional file 2: Table S5), underlining the need for taking into account the type of approach in the comparison of numerical data. Importantly, our comparison showed that relationship between the infection groups essentially remained the same, thus our conclusion appeared robust.

When comparing RSV and Influenza, a study from the US [23] published in 2019 reported higher morbidity and mortality in RSV patients. On average, RSV patients were more likely to have congestive heart failure and COPD, and they had a higher risk for ICU admission. Our findings (see Table 2, Table 3, Additional file 2: Table S5) are in accordance with this, but additionally place SARS-CoV-2 at an intermediate position between the other two viruses, with more similarities to RSV than to Influenza A.

In order to identify differences between viruses in the risk profile of patients in the most recent season, we included a comprehensive analysis of comorbidities in adults. Consistent with previous reports [21, 23], adults hospitalized with RSV had the greatest frequency of comorbidities, especially heart failure, rheumatic disease, COPD and asthma, as well as the status of immunosuppression. In the SARS-CoV-2 group, only PAD was more frequent. The higher frequency of comorbidities in RSV patients was supported by the higher median of the sum of comorbidities. Among adults, the SARS-CoV-2 group was the oldest on average, followed by RSV and Influenza A. As comorbidities are often linked to age, we assessed whether the different patterns of comorbidities were due to the differences in age. This was not the case, suggesting intrinsic age-independent risk profiles for the three infections.

The respiratory symptoms cough and dyspnoea were most prevalent in RSV patients, followed by Influenza A, and lowest with SARS-CoV-2. These observations are in line with results of a comparison covering different seasons [21]. Taken together with the higher frequency of obstructive airway diseases in RSV patients, the findings point at RSV but not SARS-CoV-2 as being primarily associated with clinical affectation of the lung.

While the primary outcomes ICU admission and mortality did not show marked differences between the three major infection groups in adults, we observed differences regarding the frequency of NIV, of low-flow oxygen supply during hospital stay and of oxygen supply upon admission. The highest percentages were found in the RSV group, the lowest in the SARS-CoV-2 group. The median length of the hospital stay was similar between groups, but patients infected with SARS-CoV-2 showed a right skewed distribution and based on this a statistically significantly longer hospital stay compared to RSV patients.

The overall pattern of differences between infections had two aspects. First, the burden from respiratory impairments appeared to be highest with RSV, underlining previous findings that RSV remains to be a serious concern in adults, particularly in the elderly and those with pre-existing medical conditions [21, 23–25]. In comparison, SARS-CoV-2 and Influenza A appeared to have a more systemic impact. Despite these differences, we could identify common risk factors in a comprehensive analysis of ICU admission and mortality. The strongest predictor for both outcomes was oxygen supplementation upon admission, while comorbidities did not play a role, except malignant disease for ICU admission. Regarding ICU, vital parameters were also relevant. For SARS-CoV-2, renal function played an important role, in accordance with our previous findings [18, 19, 26].

It demonstrated the overwhelming role of the requirement for initial oxygen supply for later ICU admission, and in addition younger age, male sex, malignant diseases, increased heart rate, decreased body temperature, and lower oxygen saturation. The predictors of higher in-hospital mortality were slightly different. The role of initial oxygen supplementation was confirmed, but at the same time a reduction in eGFR played a role, while reduced oxygen saturation showed only a tendency.

One of the advantages of our study may be that we covered the whole spectrum of patients’ ages and that the population of hospitalized patients comprised a large number of children and adolescents, in whom the dominant role of RSV was clearly visible. This was reflected by the findings regarding prevalence of infection, length of stay, admission to ICU, as well as high-flow and low-flow oxygen therapy. When restricting the analysis to young children of age less than 3 years, essentially the same results were obtained as for the total group of children and adolescents. We did not analyse the data from children and adolescents further, as RSV has already been discussed in many publications.

This retrospective study has a number of limitations. First, it does not allow causal inferences but only provides associations. In particular, we could not address the potential interaction between infection and severity of comorbidities beyond the regression analyses performed. Moreover, it is a single-centre study, and there is no guarantee that our findings must be valid for other regions. However, they are well compatible with historical data, suggesting their validity. Moreover, data limited to hospitalized patients cannot quantify the overall burden from the infections; for this, epidemiological studies are needed. As a strength, however, this limitation allowed for the collection of a large set of high-quality data in a well-defined population. A further advantage was that information from previous waves of Covid-19 from the same region was available for comparison [18, 19, 26]. A noteworthy limitation was the fact, that hospitalized patients were routinely tested for SARS-CoV-2 upon admission, while the (combined) testing for Influenza A/B and RSV was performed only in case of clinical hints on their potential involvement, both upon admission and during the stay. In case of any uncertainties, however, these hints were taken seriously and the appropriate tests were performed, thus in the sensitivity analysis we not only excluded patients of the SARS-CoV-2 group. Importantly, the sensitivity analysis demonstrated that differences in the indications of tests did not play a role regarding the relationship between infections. The only relevant bias might have been that patients with Influenza A or RSV infection were not recognized and not tested at all but this was unlikely due to the broad indication for testing. Due to incomplete data on patients after discharge from hospital, partly caused by legal issues in Germany, mortality referred to in-hospital mortality and may not reflect the overall mortality of viral infections. Based on previous analyses [18, 19, 26], however, we have reason to assume that mortality after discharge from the RoMed hospital did not play a significant role for the comparative analysis.

## Conclusion

In conclusion, the comparison of infections with Influenza, SARS-CoV-2 and RSV in the same season 2022/2023 in hospitalized patients showed no major differences in the rates of ICU admissions and mortality of adult patients. Overall, SARS-CoV-2 was most frequent. The adult RSV group had the highest frequencies of comorbidities, especially obstructive airway diseases, and of respiratory symptoms. Moreover, the need for oxygen supply, appearing as unfavourable indicator, was most frequent for RSV, both in adults and in children/adolescents. The data indicate a tendency for relatively higher disease burden from RSV compared to Influenza and SARS-CoV-2 in all age groups and suggest that it could be worthwhile to consider vaccination against RSV particularly in the elderly.

### Supplementary Information

**Additional file 1: Figure S1**: Changes in the distribution of infection types over time. Panel A: all ages, Panel B: patients aged <18 years, Panel C: patients aged ≥18 years.

**Additional file 2: Table S1**. Primary outcome data for all patients and all infections. Numbers (percentages) are given. For the results of statistical comparisons, see text. ICU = Intensive Care Unit. **Table S2**: Treatment characteristics for patients of age <3 years for the three major infection groups. Numbers (percentages) and median values and quartiles are given. * To account for the low case numbers, the groups Influenza A and SARS-CoV-2 were pooled and tested against RSV using Fisher’s exact test. Durations refer to the subgroups of patients in whom the respective treatment was applied. **Table S3**: Distribution of symptoms of patients aged ≥18 years for the three major infection groups. Numbers (percentages) are given. Statistical comparisons were performed using Chi-square statistics. **Table S4**: Data upon admission of patients aged at least 18 years (*with oxygen supply if present). Median values (quartiles) are given. Statistical comparisons were performed with the Kruskal–Wallis test. pO2 = arterial pressure of oxygen, pCO2 = arterial pressure of carbon dioxide, eGFR = estimated glomerular filtration rate, CRP = C-reactive protein. **Table S5**: Outcome data for patients ≥18 years of age, stratified according to the decision whether the infection likely to be causally linked to their hospital admission (right panel) or not (left panel). Mortality refers to in-hospital mortality. ICU = Intensive Care Unit.

## Data Availability

The datasets analysed during the current study are not publicly available due to patients’ privacy but are available from the corresponding author on reasonable request.

## Abbreviations

BMI: Body mass index
CAD: Coronary artery disease
CKD: Chronic kidney disease
COPD: Chronic obstructive pulmonary disease
CRP: C-reactive protein
Ct: Cycle threshold
eGFR: Estimated glomerular filtration rate
ICU: Intensive care unit
LDH: Lactate dehydrogenase
MIV: Invasive mechanical ventilation
NIV: Non-invasive ventilation
NT-proBNP: N-terminal pro b-type natriuretic peptide
PAD: Peripheral arterial disease
pCO_2_: Carbon dioxide
PCR: Polymerase chain reaction
pO_2_: Arterial pressures of oxygen
ROC: Receiver operator characteristics
RSV: Respiratory Syncytial Virus
SpO_2_: Peripheral oxygen saturation
