# Supplementary material for: Characteristics and outcomes of patients hospitalized for infection with influenza, SARS-CoV-2 or respiratory syncytial virus in the season 2022/2023 in a large German primary care centre
Source: Eur J Med Res. 2023 Dec 6;28:568. doi: 10.1186/s40001-023-01482-z (PMC10699044; doi:10.1186/s40001-023-01482-z)
Supplement: Supplementary file 1 — Additional file 1: Figure S1: Changes in the distribution of infection types over time. Panel A: all ages, Panel B: patients aged <18 years, Panel C: patients aged ≥18 years. [file 40001_2023_1482_MOESM1_ESM.pptx]

## Slide 1
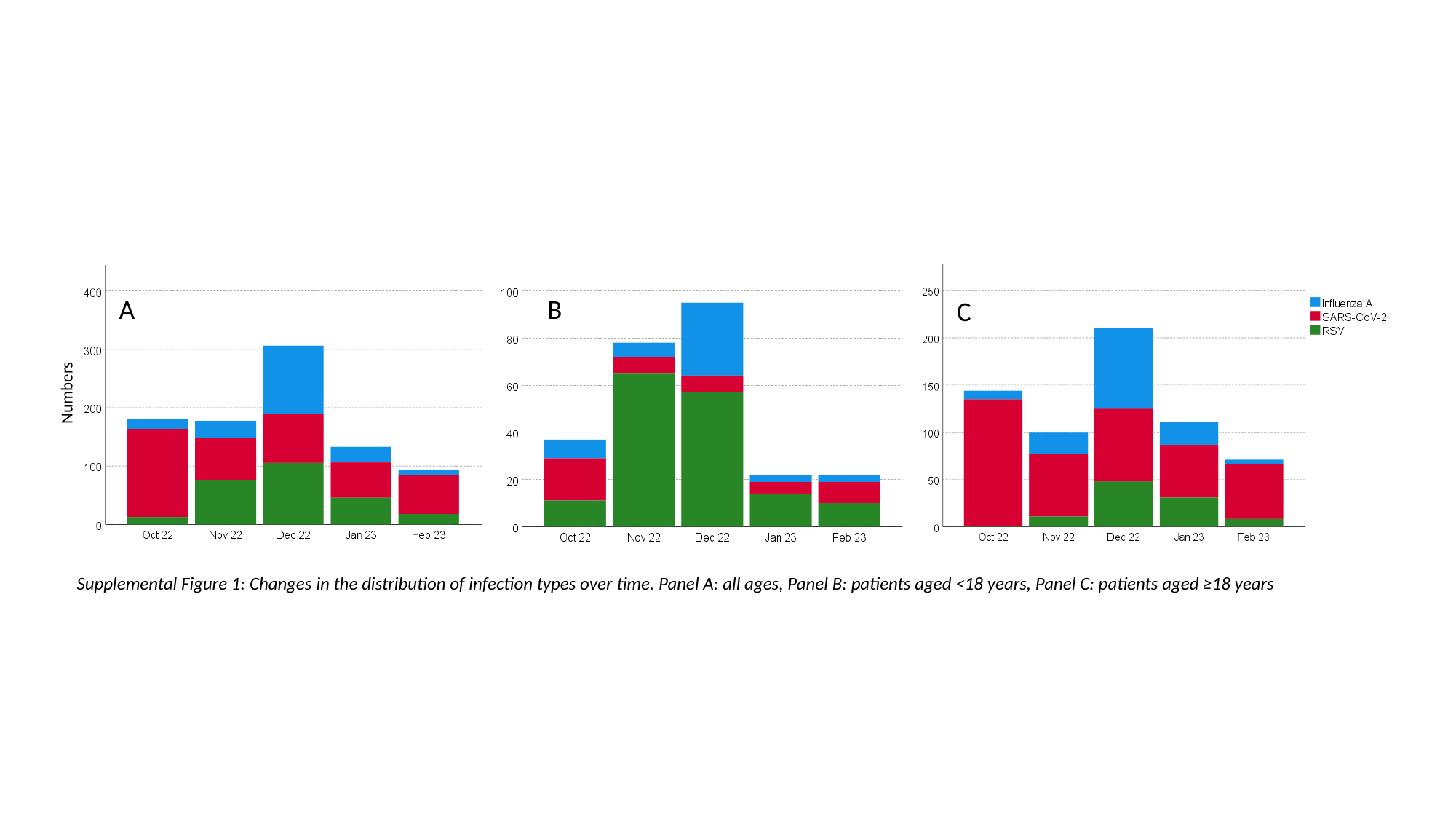

A
B
C
Numbers
Supplemental Figure 1: Changes in the distribution of infection types over time. Panel A: all ages, Panel B: patients aged <18 years, Panel C: patients aged ≥18 years
