# Supplementary material for: Characteristics and outcomes of patients hospitalized for infection with influenza, SARS-CoV-2 or respiratory syncytial virus in the season 2022/2023 in a large German primary care centre
Source: Eur J Med Res. 2023 Dec 6;28:568. doi: 10.1186/s40001-023-01482-z (PMC10699044; doi:10.1186/s40001-023-01482-z)
Supplement: Supplementary file 2 — Additional file 2: Table S1. Primary outcome data for all patients and all infections. Numbers (percentages) are given. For the results of statistical comparisons, see text. ICU = Intensive Care Unit. Table S2: Treatment characteristics for patients of age <3 years for the three major infection groups. Numbers (percentages) and median values and quartiles are given. * To account for the low case numbers, the groups Influenza A and SARS-CoV-2 were pooled and tested against RSV using Fisher’s exact test. Durations refer to the subgroups of patients in whom the respective treatment was applied. Table S3: Distribution of symptoms of patients aged ≥18 years for the three major infection groups. Numbers (percentages) are given. Statistical comparisons were performed using Chi-square statistics. Table S4: Data upon admission of patients aged at least 18 years (*with oxygen supply if present). Median values (quartiles) are given. Statistical comparisons were performed with the Kruskal–Wallis test. pO2 = arterial pressure of oxygen, pCO2 = arterial pressure of carbon dioxide, eGFR = estimated glomerular filtration rate, CRP = C-reactive protein. Table S5: Outcome data for patients ≥18 years of age, stratified according to the decision whether the infection likely to be causally linked to their hospital admission (right panel) or not (left panel). Mortality refers to in-hospital mortality. ICU = Intensive Care Unit. [file 40001_2023_1482_MOESM2_ESM.docx]

**Supplement**

**Characteristics and outcomes of patients hospitalized for infection with Influenza, SARS-CoV-2 or Respiratory Syncytial Virus in the season 2022/2023 in a large German primary care center**

Quarg C, Jörres RA, Engelhardt S, Alter P, Budweiser S

**Supplemental Results**

**Changes in infection types over time**

The distribution of the three major infection types as illustrated in the Supplemental Figure 1A significantly changed over time (p<0.001). The relative prevalence of RSV was highest from November 2022 to January 2023, while that of SARS-CoV-2 was reduced in these months. Influenza A had its peak in December 2022. Changes over time were also seen, when analysing the data from patients <18 years of age and of ≥18 years of age separately (p<0.001 each). In the group <18 years, the increase in RSV and the concomitant reduction in SARS-CoV-2 prevalence was particularly obvious (Supplemental Figure 1B). In the group aged ≥18 years, there was a rise in RSV and drop in SARS-CoV-2 frequency from November to January (Supplemental Figure 1C), but in contrast to younger patients, SARS-CoV-2 remained high and Influenza A became dominant in December 2022.

**Comprehensive analysis of risk factors for outcomes**

*ICU admission*

In all analyses, the type of infection was included as predictor, irrespective of statistical significance. First, age and sex were introduced as predictors. Both were significantly (p<0.05 each) associated with ICU admission, whereas the type of infection was not. In the next step, the comorbidities COPD, asthma, heart failure, CAD, PAD, diabetes mellitus type 2, malignant diseases and rheumatic diseases were added as predictors. Active malignant diseases as well as sex showed a tendency to be associated with ICU stay (p<0.10 each). Age remained significant (p=0.001). Keeping age, sex and malignant diseases as predictors, the vital signs upon admission shown in the Supplemental Table S4 were added as predictors. Age, sex, malignant disease, respiratory rate, heart rate, body temperature and peripheral oxygen saturation remained significant (p<0.05 each). When these predictors were retained and eGFR and oxygen supply upon admission were added, age, sex, malignant diseases, heart rate, body temperature, oxygen saturation and initial oxygen supply showed a significant relationship to ICU admission. The statistical robustness of these predictors was confirmed by stepwise forward and backward selection. Noteworthy, the type of virus was never statistically significant, suggesting that the predictors identified applied to all three types of infections. The odds ratios and their 95% confidence intervals regarding ICU admission are illustrated in Figure 2.

*In-hospital mortality*

Again, in all analyses, the type of infection was included as predictor, irrespective of statistical significance. First, age and sex were introduced as predictors. Only age remained significant (p=0.022), while sex (p=0.959) and the type of infection were not associated. When adding the comorbidities COPD, asthma, heart failure, CAD, PAD, diabetes mellitus type 2, malignant disease and rheumatic disease as predictors to age, only age remained significant (p =0.042), while all other variables showed p-values >0.10. Keeping age as predictor, the vital parameters shown in the Supplemental Table S4 were added. A significant relationship was found for age, oxygen saturation and systolic blood pressure (p<0.05 each). After adding eGFR and initial oxygen supply as predictors, both of them were significant (p<0.05 each), while oxygen saturation upon admission and systolic blood pressure showed a tendency of association (p<0.10 each). Age was no more relevant. When performing stepwise forward and backward selection, eGFR and initial oxygen supply as well as oxygen saturation were conformed as significant (p<0.05 each). The type of infection was never significantly related to in-hospital death. The coefficients of the final model are illustrated in Figure 3.

**Comparison of SARS-CoV-2 findings with previous results from RoMed hospitals**

In a previous analysis of SARS-CoV-2-associated mortality from the same region February to May 2021 (26), we had found values of 70 years for age, 60 ml/min for eGFR and 90% for oxygen saturation as best cut-off values related to mortality, as well as a Ct value (cycle threshold) of 26, in addition to initial oxygen supplementation. To assess whether these values had changed over time, we determined optimal (Youden criterion) cut-off values in the group of all SARS-CoV-2 patients. The most adequate value for age was 83.5 years, i.e. higher than the previously found value of 70 years, which was worthless in the present population. Regarding eGFR, the best cut-off value was 52.5 ml/min, which was lower than the previously found value. Regarding oxygen saturation, the best value was 94%, which was higher than the previously found value. The Ct value was not significantly associated with in-hospital mortality; the best cut-off value appeared to be in the range of 20.

Supplemental Discussion

To directly compare risk factors for mortality with results obtained in the same hospital in February to May 2021 (26), we performed a separate analysis only for SARS-CoV-2. For a direct comparison and re-evaluation, we also included the PCR Ct value. When comparing the optimal cut-off values between the previous and the present data, it appeared that mortality risk was now associated with more advanced age but no more with the Ct value. Compared to the first half of 2021, it also seemed that a higher burden from the systemic risk factor renal function was required for an increased mortality risk. The role of oxygen saturation for the primary outcomes was always difficult to evaluate in our study, as in many patients we did not have values assessed without oxygen supplementation. The results might be due to less aggressive SARS-CoV-2 variants, as indicated by the lack of association between Ct values and mortality, while at the same time higher age and more impaired renal function suggested a higher systemic burden to be required for increased mortality risk.

**Legends to Supplemental tables and figures**

**Additional file Table S1:**
Primary outcome data for all patients and all infections. Numbers (percentages) are given. For the results of statistical comparisons, see text. ICU = Intensive Care Unit

**Additional file Table S2:**
Treatment characteristics for patients of age <3 years for the three major infection groups. Numbers (percentages) and median values and quartiles are given. * To account for the low case numbers, the groups Influenza A and SARS-CoV-2 were pooled and tested against RSV using Fisher’s exact test. Durations refer to the subgroups of patients in whom the respective treatment was applied.

**Additional file Table S3:**Distribution of symptoms of patients aged ≥18 years for the three major infection groups. Numbers (percentages) are given. Statistical comparisons were performed using chi-square statistics.

**Additional file Table S4:**
Data upon admission of patients aged at least 18 years (*with oxygen supply if present). Median values (quartiles) are given. Statistical comparisons were performed with the Kruskal-Wallis test. pO2 = arterial pressure of oxygen, pCO2 = arterial pressure of carbon dioxide, eGFR = estimated glomerular filtration rate, CRP = C-reactive protein

**Additional file Table S5:**
Outcome data for patients ≥18 years of age, stratified according to the decision whether the infection likely to be causally linked to their hospital admission (right panel) or not (left panel). Mortality refers to in-hospital mortality. ICU = Intensive Care Unit

**Supplemental Figure 1:**Changes in the distribution of infection types over time. Panel A: all ages, Panel B: patients aged <18 years, Panel C: patients aged ≥18 years

**Supplemental Tables**

| **Primary outcomes** | | | |
| --- | --- | --- | --- |
| **Viral infection** | **n** | **ICU admission** | **In-hospital mortality** |
| **Influenza A** | 198 | 26 (13.1%) | 10 (5.1%) |
| **Influenza B** | 2 | 1 (50%) | 0 (0%) |
| **SARS-CoV-2** | 437 | 60 (13.7%) | 35 (8%) |
| **RSV** | 256 | 34 (13.3%) | 11 (4.3%) |
| **Influenza A + SARS** | 4 | 1 (25%) | 0 (0%) |
| **Influenza A + RSV** | 6 | 0 (0%) | 0 (0%) |
| **Influenza B + RSV** | 1 | 0 (0%) | 0 (0%) |
| **SARS + RSV** | 2 | 0 (0%) | 0 (0%) |

Supplemental Table S1: Primary outcome data for all patients and all infections. Numbers (percentages) are given. For the results of statistical comparisons, see text. ICU = Intensive Care Unit

| **Treatment characteristics of patients aged <3 years** | | | | |
| --- | --- | --- | --- | --- |
|  | **Influenza A** | **SARS-CoV-2** | **RSV** | **p value** |
| **n** | 19 | 38 | 132 | - |
| **Length of hospital stay, days** | 2 (1; 6) | 2 (1; 3) | 4 (2; 6) | <0.001 |
| **Intensive care unit** |  |  |  |  |
| Frequency | 1 (5.3%) | 0 (0%) | 11 (8.3%) | - |
| Length of stay, days | 7.8 | - | 4.8 (2.4; 12.6) | - |
| **Mechanical invasive ventilation** |  |  |  |  |
| Frequency | 0 (0%) | 0 (0%) | 2 (1.5%) | - |
| Duration, hours | - | - | 171.3 (n.d.) | - |
| **Non-invasive ventilation** |  |  |  |  |
| Frequency | 0 (0%) | 0 (0%) | 2 (1.5%) | - |
| Duration, hours | - | - | 66.9 (n.d) | - |
| **Oxygen supplementation** |  |  |  |  |
| High-flow during stay | 1 (5.3%) | 1 (2.6%) | 24 (18.2%) | 0.006* |
| Low-flow during stay | 8 (42.1%) | 0 (0%) | 95 (72.0%) | <0.001* |
| Upon admission | 1 (5.3%) | 0 (0%) | 9 (6.8%) | - |

Supplemental Table S2: Treatment characteristics for patients of age <3 years for the three major infection groups. Numbers (percentages) and median values and quartiles are given. * To account for the low case numbers, the groups Influenza A and SARS-CoV-2 were pooled and tested against RSV using Fisher’s exact test. Durations refer to the subgroups of patients in whom the respective treatment was applied.

| **Prevalence of symptoms of patients aged ≥18 years** | | | | |
| --- | --- | --- | --- | --- |
|  | **Influenza A** | **SARS-CoV-2** | **RSV** | **p value** |
| **n** | 147 | 391 | 99 | - |
| **Cough** | 91 (61.9%) | 153 (39.1%) | 75 (75.8%) | <0.001 |
| **Dyspnoea** | 79 (53.7%) | 133 (34.0%) | 72 (72.7%) | <0.001 |
| **Fever** | 83 (56.5%) | 135 (34.5%) | 38 (38.4%) | <0.001 |
| **Diarrhoea** | 8 (5.4%) | 33 (8.4%) | 7 (7.1%) | 0.493 |
| **Nausea** | 15 (10.2%) | 55 (14.1%) | 13 (13.1%) | 0.495 |
| **Fatigue** | 112 (76.2%) | 300 (76.7%) | 78 (78.8%) | 0.884 |

Supplemental Table S3: Distribution of symptoms of patients aged ≥18 years for the three major infection groups. Numbers (percentages) are given. Statistical comparisons were performed using chi-square statistics.

| **Vital parameters, arterial blood gas and laboratory parameters at admission in patients aged ≥18 years** | | | | |
| --- | --- | --- | --- | --- |
|  | **Influenza A** | **SARS-CoV-2** | **RSV** | **p value** |
| **n** | 147 | 391 | 99 | - |
| **Respiratory rate (/min)** | 20 (17; 24) | 19 (16; 23) | 20 (17; 26) | 0.007 |
| **Heart rate (/min)** | 89 (76; 105) | 85 (70; 97) | 90 (78; 107) | 0.001 |
| **Temperature (°C)** | 37.7 (36.8; 38.6) | 36.9 (36.6; 37.8) | 37.1 (36.7; 38.2) | <0.001 |
| **Oxygen saturation (SpO_2_)* (%)** | 95 (92; 97) | 96 (94; 98) | 95 (93; 96) | <0.001 |
| **Blood pressure systolic (mmHg)** | 136 (120; 150) | 135 (118; 152) | 141 (121; 154) | 0.618 |
| **Blood pressure diastolic (mmHg)** | 78 (70; 88) | 78 (68; 88) | 80 (75; 90) | 0.048 |
| **pH** | 7.41 (7.37; 7.44) | 7.43 (7.4; 7.46) | 7.41 (7.36; 7.44) | <0.001 |
| **pO_2_ (mmHg)** | 69.1 (60.2; 88.4) | 70.0 (61.3; 84.3) | 74.9 (62.9; 88.5) | 0.245 |
| **pCO_2_ (mmHg)** | 36.3 (32.3; 41.3) | 35.2 (31.2; 39.4) | 39.0 (34.6; 44.9) | <0.001 |
| **eGFR (ml/min)** | 66.0 (45.0; 86.3) | 59.0 (37.0; 78.0) | 55.0 (38.0; 79.0) | 0.013 |
| **CRP (mg/dl)** | 3.76 (1.41; 8.06) | 2.60 (0.68; 7.07) | 2.24 (0.89; 5.70) | 0.026 |
| **D-dimers (µg/l)** | 940 (650; 2265) | 1060 (540; 2530) | 590 (370; 1420) | 0.031 |

Supplemental Table S4: Data upon admission of patients aged at least 18 years (*with oxygen supply if present). Median values (quartiles) are given. Statistical comparisons were performed with the Kruskal-Wallis test. pO_2_ = arterial pressure of oxygen, pCO2 = arterial pressure of carbon dioxide, eGFR = estimated glomerular filtration rate, CRP = C-reactive protein

| **Stratified outcomes for patients ≥18 years of age** | | | | | | |
| --- | --- | --- | --- | --- | --- | --- |
| **Causality** | **Not likely** | | | **likely** | | |
| **Infection** | n | ICU admission | Mortality | n | ICU admission | Mortality |
| **Sample size** | 202 | 38 (18.8%) | 10 (5.0%) | 435 | 65 (14.9%) | 46 (10.6%) |
| **Influenza A** | 30 | 6 (20.0%) | 0 (0%) | 117 | 18 (15.4%) | 10 (8.5%) |
| **SARS-CoV-2** | 156 | 28 (17.9%) | 9 (5.8%) | 235 | 32 (13.6%) | 26 (11.1%) |
| **RSV** | 16 | 4 (25.0%) | 1 (6.3%) | 83 | 15 (18.1%) | 10 (12.0%) |

Supplemental Table S5: Outcome data for patients ≥18 years of age, stratified according to the decision whether the infection likely to be causally linked to their hospital admission (right panel) or not (left panel). Mortality refers to in-hospital mortality. ICU = Intensive Care Unit
